# Exploring the Communication of the SASP: Dynamic, Interactive, and Adaptive Effects on the Microenvironment

**DOI:** 10.3390/ijms241310788

**Published:** 2023-06-28

**Authors:** Joëlle Giroud, Inès Bouriez, Hugo Paulus, Albin Pourtier, Florence Debacq-Chainiaux, Olivier Pluquet

**Affiliations:** 1 Laboratory of Biochemistry and Cell Biology (URBC), Namur Research Institute for Life Sciences (NARILIS), University of Namur, 5000 Namur, Belgium; joelle.giroud@unamur.be (J.G.); ines.bouriez@unamur.be (I.B.); hugo.paulus@student.unamur.be (H.P.); 2 University of Lille, CNRS, Inserm, Pasteur Institute of Lille, UMR9020-U1277-CANTHER-Cancer Heterogeneity Plasticity and Resistance to Therapies, 59000 Lille, France; albin.pourtier@univ-lille.fr

**Keywords:** ageing, senescence, intercellular communication, senescence-associated secretory phenotype (SASP), microenvironment, age-related disease, senomorphics

## Abstract

Cellular senescence is a complex cell state that can occur during physiological ageing or after exposure to stress signals, regardless of age. It is a dynamic process that continuously evolves in a context-dependent manner. Senescent cells interact with their microenvironment by producing a heterogenous and plastic secretome referred to as the senescence-associated secretory phenotype (SASP). Hence, understanding the cross-talk between SASP and the microenvironment can be challenging due to the complexity of signal exchanges. In this review, we first aim to update the definition of senescence and its associated biomarkers from its discovery to the present day. We detail the regulatory mechanisms involved in the expression of SASP at multiple levels and develop how SASP can orchestrate microenvironment modifications, by focusing on extracellular matrix modifications, neighboring cells’ fate, and intercellular communications. We present hypotheses on how these microenvironmental events may affect dynamic changes in SASP composition in return. Finally, we discuss the various existing approaches to targeting SASP and clarify what is currently known about the biological effects of these modified SASPs on the cellular environment.

## 1. Introduction

Cellular senescence was first described as a stable and irreversible cellular state, in which cells permanently stop proliferating while remaining metabolically active. Since then, our understanding and definition of cellular senescence and its various roles have constantly evolved. Currently, senescence is presented as a complex and partially heterogeneous phenotype that can occur in response to the exhaustion of the proliferative capacity of the cell, and/or as a result of exposure to intrinsic and extrinsic stressors [1,2,3,4,5]. 

Since there is no universal marker for senescence, a combination of specific biochemical markers and phenotypic features is necessary to identify senescent cells. However, there is no consensus on the number and type of markers required to identify senescent cells, as one senescent cell is not equivalent to another [6]. Despite this, multiple pieces of evidence in the literature suggest that senescent cells share several characteristics, including a strong or prolonged growth arrest, an altered metabolism [3,4], and a specific senescence-associated secretory phenotype (SASP) [7,8,9,10]. Although SASP is a hallmark shared by various types of senescence, it is heterogeneous and can evolve in conjunction with changes in gene and protein expression, impacting biochemical features, trafficking, and intercellular signaling [3]. The diversity and the dynamic nature of SASP make it a complex process to understand, which is well demonstrated by its close association with both beneficial and detrimental effects depending on the physiological context [1,11,12,13,14,15]. 

These diverse effects are commonly linked with key SASP proteins whose secretion is increased in different senescence models. However, the variable component of SASP, which is modulated according to context, is often neglected in current knowledge, despite its importance.

Here, we first present the complexity of the senescence phenotype and the new biomarkers that have recently been revealed. Then, we describe the heterogeneity and plasticity of SASP composition, as well as its different levels of regulation, and highlight its contribution to both ageing and cancer. Additionally, we emphasize the importance of further investigating the complex crosstalk between the microenvironment and the SASP. Finally, we discuss how controlling SASP might be a suitable approach to treating age-related diseases.

### 1.1. The Senescent Phenotype: A Large Definition

Cellular senescence was identified more than 60 years ago by Hayflick and Moorhead who observed in vitro that normal human fibroblasts grown in optimal culture conditions have a limited proliferative capacity [16]. Nowadays, the “Hayflick limit” is widely accepted, especially since the discovery of telomere shortening-induced genomic instability [17]. This type of senescence is therefore referred to as replicative senescence (RS). 

In recent years, progress has been made to suggest that senescence could be an adaptive stress response, resulting in the persistence of irreparable damages and mainly in a prolonged cell division arrest. Indeed, senescence can be induced by the activation of oncogenes or repression of tumor suppressor genes, known as oncogene-induced senescence (OIS) [18], or by repeated exposures to oxidative or genotoxic stress, known as stress-induced premature senescence (SIPS) [19]. Since the range of potential senescence-inducing stresses is wide, it is commonly labeled based on the nature of the inducer such as irradiation-induced senescence (IRIS) or therapy-induced senescence (TIS). One common feature of these different senescence inducers is the generation of irreparable DNA damage at the origin of the sustained cell cycle arrest. However, senescence induction can occur independently of DNA damage, such as senescence induced by sodium butyrate, a histone deacetylase inhibitor (HDACi) [20], or by mitochondrial dysfunction, known as MiDAS (Mitochondrial Dysfunction-Associated Senescence), which is associated with decreased NAD+/NADH ratios [21]. Finally, cellular senescence can also be triggered by epigenetic modifications, perturbed proteostasis, and autophagy impairment [22]. 

### 1.2. Biomarkers and Characteristics of Senescence

**Figure 1:**
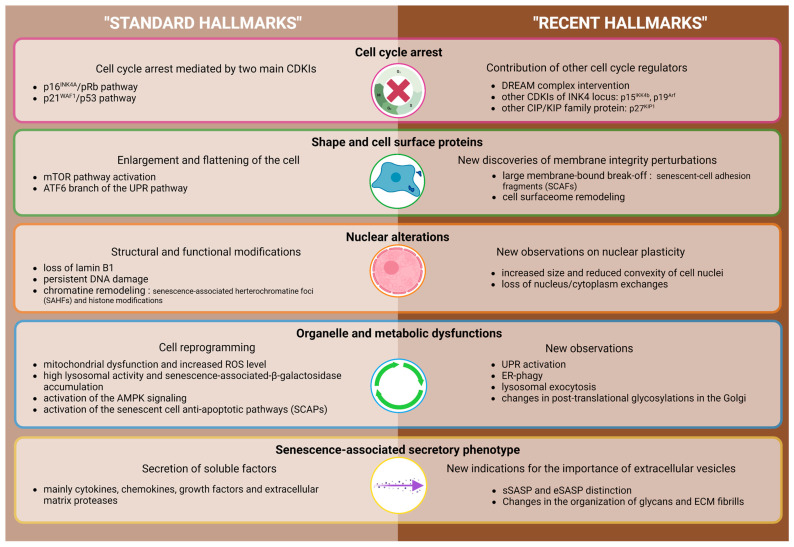
The updating hallmarks of senescence. The scheme compiles the major hallmarks of senescent cells, which are classified into five characteristic groups: cell cycle arrest, shape and cell surface proteins, nuclear alterations, organelle and metabolic dysfunctions, and senescence-associated secretory phenotype. The light and dark brown portions indicate the well-described standard hallmarks and the more recent ones, respectively. CDKIs, cyclin-dependent kinase inhibitors; UPR, unfolded protein response; ER, endoplasmic reticulum; ERAD, ER-associated degradation. This figure was created using BioRender.com.

The scientific community has faced challenges in identifying robust and specific markers that characterize the senescent state, likely due to the heterogeneity of cellular senescence. While growth arrest is the main characteristic of senescent cells, it is not sufficient to distinguish them from other non-proliferative cell states, such as quiescence or terminal differentiation. Interestingly, prolonged quiescence induces a lack of response to proliferative stimuli and progressively leads to senescence [23]. Nevertheless, the establishment of the senescent phenotype is accompanied by a set of features and alterations that are now accepted as “standard hallmarks” (Figure 1).

To ensure the senescent state of a cell, several of these hallmarks must be validated in combination.

#### 1.2.1. Standard Hallmarks

As displayed in Figure 1, the cell cycle arrest that characterizes the senescent cell often depends on the activation of two main pathways, p53/p21^WAF1^ and/or p16^INK4^/pRb. Senescent cells are enlarged and adopt a flat shape and their nuclei undergo structural and functional alterations [3]. These alterations include a decreased expression of lamin B1, a structural protein of the *nuclear lamina*, impairment of their structural integrity due to epigenetic alterations, and the formation of condensed heterochromatin regions, termed senescence-associated heterochromatic foci (SAHF) [24,25,26,27]. Senescent cells also exhibit alterations in histones (de)methylations and persistent DNA damage [28,29]. Their metabolic profile is deregulated, mainly through mitochondrial dysfunction, increased production of reactive oxygen species, elevated lysosomal activity (resulting in the SA-βgal activity), increased autophagy, and activation of the AMPK signaling pathway [30]. Apoptosis resistance via the upregulation of ephrins and anti-apoptotic proteins of the Bcl-2 family is also used as a senescence biomarker [31]. Finally, senescent cells have a particular secretory phenotype including soluble pro-inflammatory factors as well as growth factors, regulatory components of the MEC, bioactive lipids, and extracellular vesicles referred to as the senescence-associated secretory phenotype and further developed in Section 2. 

While these biomarkers are commonly encountered in the literature, it is not unusual to observe variations in their expression and effectors. 

#### 1.2.2. Other Pathways Involved in the Senescence-Mediated Cell Cycle Arrest

The cell cycle arrest mediated by the p53, p21^WAF1^, and p16^INK4^ results in the hypophosphorylation of pRb. This leads to the subsequent sequestration of E2F and prevents the expression of genes necessary for cell cycle progression. Interestingly, the p53/p21^WAF1^ pathway is also involved in assembling the repressive DREAM complex, which serves the same purpose, thereby reinforcing the role played by p53 [32,33]. However, other cell cycle regulators are also involved in this senescence-associated cell cycle arrest. Additionally, the actors of the cell cycle arrest can vary over time, with some being expressed at the onset of senescence and others during its late phases. As an example, in glyoxal-induced senescent keratinocytes, the cell cycle arrest is first mediated by the protein kinase B-FOXO3a-p27^KIP1^ pathway but is sustained over time by the p16^INK4^/pRb pathway [34]. Furthermore, in therapy-induced senescence in positive breast cancer cell lines, the tyrosine kinase inhibitor Lapatinib can induce senescence by increasing the expression of p27^KIP1^ and p15^IKK4b^ [35] and in prostate cancer cells, supraphysiological androgen levels regulate the establishment of senescence through p15^IKK4b^ [36]. Thus, it is important to extend the study of the expression of other proteins involved in cell cycle arrest and to verify their expression over time. It would be beneficial to decipher the actors involved in the cell cycle arrest and their expression patterns over time according to the cell type and senescence inducer. Currently, it is still unclear which pathways act concurrently and whether there are differences in the hierarchy and kinetics of the events, as well as which pathways are dispensable. It is worth noting that while the senescence-associated cell cycle arrest has historically been considered “irreversible”, primarily studied in fibroblasts, under certain conditions and contexts, both tumoral and normal senescent cells can resume proliferation and thus escape senescence [37,38].

#### 1.2.3. Surfaceome

Since any cell population is inherently heterogeneous, the induction of senescence only affects a fraction of the population, which therefore becomes enriched in senescent cells. Therefore, the identification, sorting, and targeting of these senescent cells using surface markers is of crucial importance. Althubiti et al. [39] were the first to perform proteomic analyses of plasma membrane-associated proteins in an EJ bladder cancer cell line with regulating expression of p21^WAF1^ or p16^INK4^. They identified 107 potential protein markers and demonstrated that a set of 10 proteins (DEP1, NTAL, EBP50, STX4, VAMP3, ARMX3, B2MG, LANCL1, VPS26A, and PLD3), when combined, could serve as markers of senescence and facilitate the detection of senescent cells in various human tissues [39]. Since this first analysis, other proteins that contribute to the surfaceome of senescent cells have been described such as the dipeptidyl peptidase 4 (DPP4) [40] and the urokinase-type plasminogen activator receptor uPAR [41]. B2MG, DPP4, and uPAR are therefore used as targets for the specific clearance of senescent cells in vitro and in vivo [41,42,43]. 

#### 1.2.4. Alterations of Nuclear Shape and Nucleus-Cytoplasm Exchanges

Senescence is also associated with alterations of the nuclear architecture. Besides the well-known modification of the *nuclear lamina* partly linked to lamin B1 loss [44], other nuclear features including nuclear matrix, nucleolus, heterochromatin, and even nuclear shape and size can also be altered [45]. In particular, the increased nuclear shape is a promising biomarker to predict the senescent state [46]. By using machine learning algorithms, the group of Heckenbach demonstrated that nuclear morphology could predict senescence following different senescent inducers, in different cell types and species. Deep learning could therefore be used as a tool to predict the occurrence of senescent cells in clinical contexts and to study their potential negative effects, paving the way for the prevention of the harmful effects of senescence [47].

Moreover, the nucleus–cytoplasm exchanges are altered in senescence, as the transcription-export (TREX) machinery and the nucleocytoplasmic trafficking (NCT) are downregulated [48,49]. Interference in exchanges between the nucleus and the cytoplasm of senescent cells leads to a reduction in the transmission of extrinsic signals toward the nucleus and alters the nucleus-to-cytoplasm protein–RNA transport, resulting in the establishment of a “nuclear barrier” [48,49].

#### 1.2.5. UPR and ER Control Quality

The endoplasmic reticulum (ER) is a dynamic structure playing a major role in the folding, synthesis, processing, and quality control of secreted and transmembrane proteins. When ER homeostasis is perturbed, an adaptive mechanism called UPR (Unfolded Protein Response) aims to restore ER homeostasis and promote cell survival [50]. It is now well accepted that different cell types undergoing senescence upon various inducers promote UPR activation [47,51]. We and other groups have shown evidence that the different arms of the UPR pathway may control several senescence hallmarks. Indeed, UPR inducers promote a premature senescence phenotype [52,53]. Moreover, the ATF6α arm of the UPR controls cell size and enlargement in replicative senescent fibroblasts [52]. In addition, the expression of autophagic component MAP1LC3-II in glioblastoma cells undergoing therapy induced-senescence is triggered by the PERK/ATF4 arm [54], and in Hras-induced senescence, production of SASP induces the UPR activation via proteotoxic stress [55,56]. The role of the UPR regarding SASP is discussed in Section 3.

How senescence affects the other ER quality control systems, namely, the ERAD (Endoplasmic reticulum-associated protein degradation) and ER-phagy, is poorly understood. To date, only one study has reported a relationship between ER-phagy and senescence. The authors demonstrated activation of the ER-phagy mediated by FAM134B, an ER-phagy receptor, upon advanced glycation end product (AGE) stress-induced senescence in primary human nucleus pulposus (NP) cells [57]. They also found that enhancement of ER-phagy by FAM134B overexpression reduces the percentage of SA-β Gal positive cells, while the suppression of FAM134B exacerbates it upon AGEs stress in NP cells [57]. The role of ERAD and ER-phagy deserves to be further explored in senescence.

## 2. Characteristics of SASP

During its lifespan, a cell can release a range of molecules into its surrounding extracellular environment. The secretory profile is constantly influenced by intrinsic characteristics, which depend on the cell type and its differentiation stage, as well as extrinsic factors such as the change in the cellular environment. Indeed, a clear remodeling of the secretory profile can be observed in cells undergoing senescence [58]. Examining the secretome of senescent cells reveals modifications in the levels of soluble, insoluble, and extracellular vesicle (EV)-related components. Senescence establishment can cause these components to be either exacerbated or partially depleted, and can also lead to the secretion of new components when compared to proliferative cells [59]. 

### 2.1. Reported SASP Factors

Senescent cells exhibit a distinct and dynamic secretome different from their exponentially growing counterparts [60]. This SASP is complex and is composed of hundreds of different proteins and non-protein signaling molecules [60,61]. Despite the diversity of the factors secreted, a core protein secretome can be distinguished from the soluble part of SASP (sSASP) (Table 1). In human primary fibroblasts subjected to various senescence inducers (IR, RAS, atazanavir (ATV)), this core protein sSASP includes, among others, STC1 (stanniocalcin 1), chemokines such as CXCL1 (C-X-C motif ligand 1), and proteases such as MMP-1 (matrix metalloproteinase 1) [60]. Hemostasis-related factors, another class of bioactive compounds, show a marked increase in secretion into the extracellular medium by senescent human primary fibroblasts when exposed to different inducers (IR, doxorubicin (DOX), and MiDAS) [61]. Non-protein signaling molecules, including various bioactive oxidized lipid metabolites, prostaglandins, and nitric oxide can also be found enriched in the sSASP of senescent cells [62,63,64,65]. While empirical research has focused on soluble factors secreted by senescent cells, new studies show evidence that EVs are also a substantial and effective part of SASP [66]. EVs are lipid membrane vesicles containing cytosol from the secreting cells and are released by multiple cell types. According to their origin, biological function, and secretion, EVs can be classified into two main subtypes: exosomes and microvesicles (MVs) [67]. In this context, it has been evidenced in multiple cell types that EV secretion increases after exposure to different senescence inducers, with changes in cargo composition including proteins and genomic content such as microRNAs (miRNAs) and lipids [63,68,69]. 

The core secretome is a concept based on a limited number of established cellular models used to study senescence, such as human primary fibroblasts, and represents only a portion of the complete picture. Hence, the application of a core SASP should be considered as a tool to evaluate the acquisition of the senescent phenotype in a standardized manner, rather than a way to generalize the effects of SASP on the cellular microenvironment. 

Indeed, the bioactive effects of SASP may be more closely linked to specific and possibly subtle variations in the secretome that result from the combination of a specific cell type and a particular senescence inducer, rather than the shared components. 

### 2.2. SASP Heterogeneity and Plasticity

Coppé and colleagues demonstrated initially that only a subset of SASP proteins was shared between fibroblasts and prostate epithelial cells upon irradiation-induced senescence (IRIS) [9]. A subsequent large-scale proteomic analysis of SASP then revealed only 58 shared SASP factors between fibroblasts and renal epithelial cells in IRIS [60]. When considering other proteomic studies on various cell types and senescence inducers, such as UVA-induced senescent keratinocytes and IRIS mesenchymal stem cells [71,72], the number of shared SASP factors drops to 19, suggesting that only a handful of proteins are commonly secreted across all types of senescent cells. Given the differences in experimental parameters such as EV isolation methods, detection techniques, and time points assessed after senescence induction, comparing these studies remains challenging. 

Secondly, some SASP factors are secreted at different times depending on the cell type. For instance, in IRIS, the *IL*-*1β* gene is overexpressed on days 10 and 20 in fibroblasts, but only on day 10 in keratinocytes, and on day 20 in melanocytes [73]. This point emphasized SASP plasticity over time. Moreover, we showed an increased secretion of IL-6 and IL-8 in UVB-induced senescent keratinocytes on day 3 that disappeared on day 7 following senescence induction [70]. In addition, HDFs in replicative senescence also exhibit two distinct phases of SASP gene expressions. The first phase is often characterized by an overexpression of SASP genes related to inflammation, such as *IL*-*6* and *IL*-*8*, while the second phase is more characterized by changes in the expression of genes associated with extracellular matrix modulation, such as *MMPs* [74]. This is related to the temporal regulation of SASP, further developed in Section 3. Further experiments are required to better understand the kinetics of SASP factors and their physiological relevance in the short and long term.

Thirdly, SASP composition is also influenced by the senescence inducers. Senescent IMR-90 fibroblasts present a different secretome profile depending on whether the senescence was induced by X-rays, ATZ, or RAS overexpression [60]. Similar results were observed on mesenchymal stem cells in senescence induced by oxidative stress, doxorubicin treatment, X-ray irradiation, or replicative exhaustion [72]. 

Finally, the matrix and cellular microenvironment of senescent cells can impact their secretome composition. The substrate stiffness impacts the NF-κB phosphorylation status in UV-induced senescence in fibroblasts [75], suggesting that the ECM composition could have an impact on SASP composition. Moreover, it has been demonstrated that co-cultivating squamous cell carcinoma and RS fibroblasts exacerbate some SASP gene expressions, such as *IL*-*8*, *IL*-*1β*, or *CCL2*, in RS fibroblasts [76]. These findings demonstrate the need of developing cellular models that consider the influence of the ECM and other cell types within the same tissue. Such models would allow a better understanding of the complex interplay between SASP and its microenvironment, which can affect SASP composition over time. 

## 3. Regulation of SASP

The regulation of SASP involves transcriptional, post-transcriptional, epigenetic, and translational mechanisms. In addition, the secretion of SASP components is regulated through intracellular trafficking, and many compartments of secretion are altered during senescence. These alterations could potentially affect the dynamic and heterogeneous composition of SASP. 

### 3.1. Transcriptional Regulation

Multiple signaling pathways have been identified to activate transcription factors that play a crucial role in regulating the expression of inflammatory cytokines. 

First, there is a clear link between the expression of SASP and the DNA Damage Response (DDR) pathway, as several DDR proteins (ATM, Chk2, and NBS1) are necessary for the initiation and maintenance of the cytokine response in IRIS fibroblasts [29]. It has recently been described that in the absence of DNA damage, such as after sodium butyrate treatment, the SASP of fibroblasts still relies on the non-canonical activation of DDR and the accumulation of ATM, MRE11, and NF-kB on chromatin [77]. However, the expression of SASP factors seems independent of the cell-cycle regulators p53 and pRb, as their invalidation or inactivation even promotes IL-6 secretion [9,29]. On the other hand, it has been recently demonstrated that the knockdown of p16 in fibroblasts decreases the expression of IL-6 and IL-8 in OIS and etoposide-induced senescence [78]. 

Then, the NF-κB and C/EBPβ transcription factors were identified to be involved in the regulation of CXCR2 ligands expression, including IL8 in fibroblasts in OIS [79]. The activation of NF-κB in fibroblasts has been demonstrated to depend on GATA4, whose regulation by p62 is suppressed during senescence but mediated by DDR-related ATM and ATR [80]. NOTCH1 plays a dual role in the regulation of SASP. It is positively associated with early SASP expression in OIS in fibroblasts but then represses late SASP expression by suppressing C/EBPβ expression [81]. 

Regarding the regulation of inflammatory cytokine expression, the activation of another signaling pathway involved in inflammation, JAK/STAT, has also been demonstrated in a PTEN-deficient prostate cancer mouse model [82], as well as in senescent preadipocytes [83]. 

Finally, the cGAS/STING pathway has been highlighted to be involved in the regulation of inflammatory SASP factors, notably, IL-6 and CXCL10 secretion, via NF-κB activation in vitro and in vivo [84], following the detection of cytoplasmic chromatin fragments (CCFs) [85] associated with a loss of nuclear integrity following Lamin B1 (*LMNB1*) downregulation [86]. It has recently been demonstrated that COX2 plays an important role in regulating the expression of several inflammatory SASP components in OIS through an autocrine feedback loop involving prostaglandin E2 (PGE2) binding to EP4, but the downstream pathways of PGE2 and EP4 remain unknown. Nevertheless, the COX2 pathway is thought to be able to activate major SASP transcriptional regulators, such as NF-κB, C/EBPβ, and GATA4 [87]. 

### 3.2. Post-Transcriptional Regulation

While early SASP is mainly regulated at the transcriptional level, its long-term SASP expression is mainly driven by post-transcriptional mechanisms. This has been demonstrated by the lack of impact of actinomycin D treatment, an inhibitor of transcription, on the expression of several SASP factors [88]. P38^MAPK^ appears to be an important factor in the temporal regulation of SASP. If it is first activated after the induction of senescence, it enables the expression of SASP factors, such as IL-6 and IL-8, through NF-κB activation in IRIS fibroblasts [89]. It is also involved in the subsequent post-transcriptional regulation of SASP by restricting the binding of AUF1 to the 3′-UTRs of several SASP mRNAs, including *IL*-*6* and *IL*-*8*, thereby preventing their destabilization, as demonstrated in bleomycin-induced senescent fibroblasts [88]. The mTOR pathway is also involved in the post-transcriptional regulation of SASP. Specifically, mTOR activates the translation of MK2 (or MAPKAPK2), which can phosphorylate and inhibit the RNA-binding protein ZFP36L1, also involved in the destabilization of several SASP mRNAs [90]. The mTORC1 kinase has also been shown to modulate senescence-induced inflammation and SASP [91]. 

As previously mentioned, studies on the regulation of SASP have primarily focused on the transcriptional and post-transcriptional regulation of inflammatory cytokines. However, there is a limited understanding of the regulatory mechanisms underlying other SASP factors, such as growth factors and proteases. A recent study on fibroblasts has described that E2F4, TEAD1, and AP-1 transcription factors are major regulators of RS [92]. Moreover, AP-1 is involved in the expression of *IL*-*6*, *IL*-*1β*, and *MMP*-*10*, as their expression is abrogated when expressing a dominant-negative isoform of c-Jun, one of the subunits of AP-1, during OIS in fibroblasts [93]. Future studies focusing on the regulation of non-inflammatory SASP factors would be valuable. 

### 3.3. Epigenetic Regulation

The physical clustering of SASP genes, such as *MMPs* (*MMP*-*1*, -*3*, -*10*, and -*12*) or chemokines (*CXCLs* and *CCLs*), suggests that the regulation of their expression may depend, at least in part, on broader changes in chromatin conformation [94]. Indeed, several histone variants can influence the expression of SASP genes. For example, the relocation of the macroH2A1 histone variant away from SASP genes following ER stress response-mediated activation of ATM in fibroblasts in OIS is involved in the maintenance of SASP gene expression [56]. Moreover, the increased expression of histone variant H2A.J in fibroblasts undergoing etoposide-induced senescence enhances the expression of multiple genes associated with inflammation and immune response. This effect is likely attributed to the interaction of H2A.J with other factors [95]. In addition, nuclear HMGBs bind to DNA, facilitating the access of transcription factors to promoter regions. In fibroblasts in RS or IRIS, HMGB1 can be released into the extracellular space and act as an alarmin to activate NF-kB, which subsequently upregulates the expression of pro-inflammatory target genes [96]. Furthermore, HMGB2 preferentially localizes to SASP gene regions during OIS in fibroblasts, protecting them from being incorporated in transcriptionally repressed SAHF regions [97].

### 3.4. Secretory Control: Compartments of Secretion and Vesicular Trafficking

Even though most organelles are morphologically or functionally affected during senescence, their proportion increases in senescent cells due to various signaling defects. In addition to nuclear and mitochondrial dysfunction, the endoplasmic reticulum, Golgi apparatus, and lysosomal compartments are strongly involved in the generation, processing, and release of SASP factors [91].

The ER is the site of membrane biosynthesis used in secretory and excretory pathways. It is responsible for folding and maturating secreted proteins, making it the first compartment of secretion. Recently, it has been proposed that ER stress and the subsequent activation of the unfolded protein response upon senescence could contribute to the modified secretome of senescent cells [98]. While there are multiple connections between the UPR and inflammation [99,100], the UPR and normal or tumoral secretome [101,102,103], as well as the UPR and direct control of MMPs [104], the data directly linking ER stress with SASP are scarce. Our group demonstrated that knocking-down ATF6 in RS fibroblasts decreased *IL*-*6* mRNA levels [57]. Chen et al. [56] proposed that UPR induction in RAS-mediated senescence led to macroH2A1 expression, which, in turn, induces the expression of various SASP-associated genes in fibroblasts. Moreover, Dorr et al. [55] suggested that OIS and TIS induce proteotoxic stress and UPR activation to ensure SASP production. Nevertheless, the exact role of ER and UPR must be further addressed to confirm a central role in the establishment and composition of SASP.

The Golgi structure is also altered in senescent cells [105]. These alterations can not only be mediated by the translocation of a G protein γ subunit from the plasma membrane to the Golgi [106] but also by the impaired expression of the vacuolar ATPase ATP6V0A2, which acidifies organelles such as Golgi, endosomes, or lysosomes [107]. This results in deep changes in post-translational glycosylation in the Golgi, impacting SASP compounds. In addition, the trans-Golgi network (TGN) appears to be increased in senescent cells. A blockade of TGN components such as the protein kinase D1 (PKD1) is associated with the intracellular accumulation of some SASP factors including IL-6 and IL8 in OIS fibroblasts [108]. 

Lysosomes are at the crossroads of endocytic and exocytic pathways, and their increased abundance in senescent cells may be associated with the exacerbation or deregulation of these pathways. Besides their partnership with the Golgi apparatus and the endosomal compartments, lysosomes are also important for the clearance of cytoplasmic chromatin fragments (CCFs). CCFs may leak from the nucleus in the cytoplasm of senescent cells and induce an SASP; both CCFs and SASP inductions would be related to a retrograde mitochondrial–nucleus signaling pathway associated with the mitochondrial increase in ROS species [91].

In melanoma cells, the lysosomal exocytosis mediated by the small GTPase RAB27A has also been shown to be upregulated in TIS and to participate in SASP factors secretion, including the chemokines CCL-2 and CXCL-12 [109]. Along with this enhanced lysosomal secretion, senescent cells exhibit a remodeling of their lysosomal proteome with selective enrichment in some lysosomal resident proteins such as those implicated in vesicular transport and fusion [109].

Small EV and exosome secretions are now part of the specific secretory phenotype. The release of senescence-associated exosomes is linked to RAB27A expression, as silencing of *RAB27A* leads to decreased exosome secretion in fibroblasts undergoing RS or OIS [110]. Rab27 GTPases are associated with the connection of multivesicular endosomes and the secretion of exosomes [111]. The enhanced biogenesis of EVs and their release by senescent cells have been demonstrated to be associated with the extent of DNA damage generated by the senescence inducer, as well as the activation of the ceramide synthetic pathway [112]. EVs and exosomes also contribute to SASP and its paracrine impact. For example, EVs from senescent stromal cells can enhance the proliferation of cancer cells by promoting the activation of the ephrin-A2 tyrosine kinase receptor, which interacts with overexpressed ephrin-A1 on the surface of the cancer cells, thereby boosting an Erk-dependent proliferation pathway [110,113]. In addition to being components of SASP, the release of senescence-associated EVs seems to be a mechanism used by senescent cells to discard cytoplasmic chromatin DNA fragments (CCFs), thus limiting DNA damage accumulation caused by major stress exposure and potentially modulating SASP [110,112]. Finally, EVs and exosomes are opening a new era of a multifunctional SASP due to their wide range of potential contents, as well as the specificities of the cellular niches and partnerships in which they operate.

## 4. Senescence and SASP In Vivo

Senescent cells accumulate in tissues with age. A meta-analysis showed that even if the proportion of senescent cells in 14 different human tissues is correlated with chronological age, it varies depending on the tissue type and the senescence marker used [114]. Moreover, the accumulation of senescent cells is also detected at pathological sites due to various stress signals regardless of age [115]. As a result, there is a wide diversity of senescent cells throughout the body. Furthermore, several studies have indicated that the elimination of senescent cells using transgenic mice, such as the INK-ATTAC and p16-3MR mouse models that both specifically target the elimination of p16-positive cells [15,116], or through the use of small pharmacological molecules called senolytics (which kill senescent cells) or senomorphics (which suppress some or all of their phenotype/properties) has shown improvements in healthspan, alleviated several age-associated conditions, delayed tumor formation, and mitigated the side effects of chemotherapy [117,118,119,120]. These findings highlight senescence as a significant contributor to ageing and associated pathologies. Therefore, SASP profiles may contribute to developing senescence biomarkers in human plasma or other biofluids, as well as assessing the efficacy of senescence-targeted therapies (see Section 6). Basisty and colleagues defined in senescent culture cells a core SASP including GDF15, STC1, SERPINE1/PAI-1, and MMP1, which are also reported to be significantly increased among the plasma markers of ageing in humans [60]. Another study showed that doxorubicin-induced senescence enriched the SERPINE1/PAI-1 SASP factor in plasma in vivo [61]. However, these markers can also serve as biomarkers for several diseases such as cardiovascular, metabolic, neurodegenerative, and malignant diseases, regardless of age. This makes them indicators of a “state of ageing” rather than a chronological accumulation of senescent cells. Surprisingly, the production of SASP factor IL-6 is increased in in vitro senescent models, but the circulating levels of IL-6 are not significantly different between young and elderly subjects [121,122]. Interestingly, Markov et al. identified, by using machine learning on a human cohort, immune biomarkers to predict brain ageing and suggested that intervention on these biomarkers could prevent brain ageing [123]. Regarding the use of senolytics for treating human cellular senescence-associated diseases, it is worth noting that the SASP factors assayed or detected can vary significantly from one study to another [124,125]. These variations are undoubtedly specific to the origin of senescent cells, which makes them difficult to use as markers in clinical settings. Further studies are needed to be able to robustly consider SASP factors, or a subset of them, as reliable biomarkers and to determine how SASP could be effectively translated into clinical applications.

## 5. Pleiotropic Roles of SASP 

Due to its diverse composition, SASP can have pleiotropic effects on the cellular environment, which can be either beneficial or deleterious. Several recent publications have extensively covered this topic [10,58,126]. However, in this section, we focus on certain physiological and pathophysiological consequences that SASP can cause. Specifically, we examine how SASP influences extracellular matrix remodeling, intercellular communication modification, ageing, and the development of cancer.

### 5.1. Extracellular Matrix Remodeling 

Collagen alterations in the dermal ECM have been associated with the decline in human skin structure and function during ageing. This emphasizes the overexpression of cysteine-rich protein 61 (CCN1) and MMP-1 expression in the SASP of senescent fibroblasts [127]. Changes in ECM composition and ECM-degrading molecules produced by SASP also disrupt elastin and collagen fiber networks and basement membranes in ageing tissue [128]. Therefore, SASP-related changes in ECM components have a notable effect on cell functions and fates by altering the tortuosity of collagen or by increasing the stiffness of the ECM as ageing progresses. 

In the context of tissue injury, senescent cells can play a role in regeneration by accelerating wound healing or limiting fibrosis. For instance, the transient secretion of PDGF-AA (Platelet-derived growth factor AA) from senescent fibroblasts is necessary for effective healing following skin injury [15]. Strikingly, the short-term presence of miR-23a-3p in EVs derived from senescent fibroblasts allows a faster wound closure of epidermal keratinocytes [129]. However, to date, few studies have investigated the link between senescent cells and ECM since the matricellular protein CCN1 has been shown to induce ROS-induced senescence in fibroblasts during wound healing [130]. In addition, the secretome of senescent HSCs (hepatic stellate cells) plays an important role in fibrotic degradation and maintenance of liver tissue homeostasis [131]. Moreover, the elimination of senescent p16^High^ LSECs (Liver Sinusoïd Endothelial Cells) in mice induces fibrosis [132]. Recently, efforts have been made to better characterize the changes in the matrisome of senescent cells and their effects on the environment. Hierbert and colleagues [133] described that the activation of Nrf2 in fibroblasts triggers the production of a senescence-promoting ECM via the expression and secretion of certain ECM proteins, such as PAI-1. This can accelerate wound closure and promote re-epithelization in vivo. In addition, Nrf2 inhibition in fibroblasts reduces the production of collagen I and alters ECM deposition [134]. Furthermore, when muscle stem cells are seeded onto decellularized ECM maintained by senescent fibroblasts, their responses and functions are affected, resulting in enhanced expression of fibrogenic markers and reduced myogenic markers [135]. 

### 5.2. Tumor Suppression and Promotion

While cellular senescence is widely recognized as an anti-tumor barrier, there is growing evidence to suggest that senescence may also have a tumor-promoting role. 

Senescent cells have been observed at sites of benign tumors, such as prostatic hyperplasia and melanocytic naevi [136,137]. The factors secreted by these cells influence the tissue microenvironment and impact cellular differentiation and proliferation, notably in cancer cells [136,138]. Moreover, the first studies pointing out the role of the cellular microenvironment in the promotion of cancer progression highlighted the role of Carcinoma-Associated Fibroblasts (CAFs) in prostate cancer progression [139]. It has been subsequently reported that senescent fibroblasts share many features with CAFs, and can have a similar impact on the differentiation of epithelial cells initiated by cancer, and on tumor growth both in vitro and in vivo [13,140]. Co-culture systems and xenograft models have shown that SASP from senescent fibroblasts promotes the tumorigenesis of premalignant epithelial cells [141], induces epithelial–mesenchymal transition (EMT), and increases tumor vascularization, which suggests pro-tumorigenic properties [98]. 

We and others have shown that Normal Human Mammary Epithelial Cells (HMECs) and Normal Human Epidermal Keratinocytes (NHEKs) can spontaneously and systematically escape from the senescent state [142]. Some rare senescent cells re-enter the cell cycle via a process called Post-Senescence Neoplastic Escape (PSNE) with characteristics similar to those observed during the early stages of tumor initiation [52,143,144,145,146]. More importantly, when xenografted in nude mice, these PSNE cells developed into disseminated skin lesions such as hyperplasia and small non-melanoma skin carcinoma, evidencing their tumorigenic potential [144,145]. SASP from senescent dermal fibroblasts promotes neoplastic escape from normal human keratinocytes and increases markers of EMT as well as the migration of emerging cells [145]. This was attributed to the activation of the membrane PAR-1/Thrombin receptor by MMPs among SASP of senescent fibroblasts [145]. Furthermore, a recent study has identified a BDNF-TrkB axis as being associated with the role of SASP of aged fibroblasts in promoting EMT initiation in primary keratinocytes from aged donors [147]. 

Another point is that the alteration in the secretion of ECM components and regulators by senescent prostate cells generates a favorable environment for tumor development [148]. UVB-induced senescent fibroblasts were shown to produce an ECM that promotes proliferative signaling pathways of preneoplastic HaCaT epidermal keratinocytes [149]. Enhanced collagen deposition has been described along breast cancer progression, with dysregulated architecture and increased reticulation via abnormal expression of lysyl oxidase and MMP-resistant collagen isoforms, contributing to carcinoma progression [150]. Moreover, increased collagen matrix stiffness has been shown to control the cell fate of normal breast cells in 3D models. The increase in collagen concentration led to the overexpression of the α6β4 integrin pair, profound disruption of the architecture from regular normal-like acini and tubes to a tumor-like mass of increasing size, and decreased differentiation [151]. 

Taken together, the combination of SASP factors responsible for the pro-tumorigenic effects remains poorly understood. However, progress has been made in defining how context-dependent (such as cellular partnerships, specific soluble proteins, or membrane receptors) can influence the effects of SASP in cancer promotion [152]. 

### 5.3. Senescence Induction and Reinforcement 

In addition, SASP primarily influences the induction and reinforcement of senescence. It is now clear that senescent cells maintain their phenotype through an autocrine positive feedback loop in which the main factors identified are cytokines such as IL-6 and IL-8 [79,153]. Similarly, the same SASP factors and many others such as TGF-β family ligands, VEGF, and chemokines such as CCL2 and CCL20 also play an important role in inducing paracrine senescence in neighboring cells [154]. The intensity of SASP can impact local homeostasis paracrine through signals that propagate the senescent state, exacerbating local stress, and inducing ROS-mediated damage in neighboring cells. This is the so-called SMS effect of SASP. Hence, conditioned media (CM) of cells exposed to UV radiations (UVA, B, and C) initiate bystander DNA damage in non-exposed neighboring cells [155]. Moreover, since the recent interest in deciphering the role of eSASP, new studies have shown the important contribution of microvesicles in the propagation of the senescent phenotype, for example, via the transfer of interferon or miRNA cargo factors [156,157].

### 5.4. Other Functions of SASP

SASP can modulate the fate of neighboring cells in several ways and can even impact the differentiation of surrounding cells. 

For example, Wiley et al. [21] have shown that CM harvested from fibroblasts whose mtDNA has been depleted (rho0) can block adipogenesis in preadipocytes but promote keratinocyte differentiation. A recent study also showed that TGF-β secreted by senescent cells can influence the differentiation of T helper cells during the response to influenza infection in mice [158]. A proteomic analysis of CM of fibroblasts in IRIS identified a role for SASP in hemostasis, platelet activation, and degranulation [61]. Moreover, transient exposure of primary mouse keratinocytes to SASP of OIS keratinocytes led to enhanced plasticity via the increased expression of stemness markers and better regenerative capacities in vivo, while long-term exposure promoted senescence, reducing regenerative stimuli [159]. Therefore, SASP may induce cellular plasticity and tissue regeneration capacities according to its intensity and duration, and may promote cellular reprogramming in neighboring cells [160]. In parallel, ECM stiffness is also of particular importance in stem cell response and can lead to considerable changes in cell signaling, shape, and differentiation status [161]. Decellularized matrices form cardiac explants of donors of various ages, differentially impacting the cell fates and functions of IPSC-derived cardiomyocytes (ICMs) cultured on top of the matrices. In fact, matrices from young donors can enhance the proliferation and functions of young ICMs, while matrices from aged donors promoted their senescence [162]. In addition, SASP can reduce muscle stem cell expansion. In damaged muscle, senescent cells altered their normal niche to create an age-related-inflamed microenvironment that impairs regeneration [163].

Finally, Saul et al. [164] conducted a bioinformatic analysis and identified a panel of genes, called SenMayo, which is enriched in elderly vs. young women. They evaluated the applicability of SenMayo across tissues and species. Using their tool, they were able to evaluate intercellular communication patterns of senescent cells with other cells in their microenvironment at the single-cell level. Overall, they showed that senescent hematopoietic and mesenchymal cells interact with neighboring cells mainly through the Macrophage Migration Inhibitory Factor (MIF) pathway. 

## 6. Developing Strategies to Block SASP or Its Specific Effects 

Given the importance of senescence in physiological processes, it is reasonable to think that there is a threshold beyond which the accumulation of senescent cells induces a microenvironment conducive to the development of pathologies via SASP. The accumulation of senescent cells can also occur when the immune system ages, altering the ability of immune cells to clear senescent cells. 

Elimination of senescent cells by senolytics demonstrated a contributive role of senescent cells in ageing and age-related diseases [165] and paved the way for the development of senotherapeutic approaches. Therefore, over the past 5 years, senotherapeutic research has emerged to slow down the ageing phenotypes. Current senotherapeutic strategies targeting senescent cells are mainly based on drugs that specifically kill senescent cells (senolytics) and components that suppress the detrimental effects of SASP without inducing senescent cell death (senomorphics, also known as senostatics) [166,167,168,169,170,171,172]. Other senotherapeutic strategies include prodrugs, protein degraders, nanocarriers, and immunotherapies [173]. It is worth noting that a recent study showed that eliminating senescent cells by using chimeric antigen receptor (CAR) T cells that specifically target senescence-specific surface antigens such as uPAR improved the survival of mice with lung adenocarcinoma and restored tissue homeostasis in a chemical-induced liver fibrosis mouse model [41]. Emerging preclinical evidence has highlighted the significant potential of these approaches [27,28,124]. However, further analyses are necessary to rule out the potential adverse effects of long-term administration. Additionally, there are ongoing efforts to evaluate combinations of senotherapies in individuals with multiple age-related diseases [174]. 

Nevertheless, in this section, we will not cover all senotherapeutic strategies, especially as excellent reviews have recently been published on senolytic developments [171,175,176,177], but rather focus on those with senomorphic activities (Table 2), based on their ability to block SASP components. 

A first strategy would consist in using neutralizing antibodies, recognizing and blocking specific surface proteins upregulated at senescence. Secretion of IL-6 has been decreased in senescent HUVECs and fibroblasts treated with anti-TNFα or anti-ephrin B2 antibodies, respectively [178,179]. Several other surface proteins are known to play a role in the regulation of SASP profiles, including SCAMP4, Notch, and CD36 [40,81,180]. However, it has not yet been reported that the use of neutralizing antibodies targeting SCAMP4, Notch, or CD36 can impact the composition of SASP and, therefore, arrive at a conclusion regarding their senomorphic properties. In a model of bleomycin-induced senescence, the secretion of certain SASP factors (including IL-6 and IL-8) can be directly inhibited with neutralizing antibodies such as those against the membrane-bound IL-1α [181]. It would be interesting to investigate the impact of other neutralizing antibodies directed against other major SASP factors such as circulating IL-1β-, IL-6, or their receptors [182], on their abilities to alter the chemical composition of SASP, impair SASP-mediated effects, and attenuate other features of senescence in different cell types. 

A second strategy would be to use pharmacological and natural compounds. Many senomorphics are polyphenols (including flavonoids, phenolic acids, lignans, and stilbenesenes) that possess antioxidant activities, but their modes of action have not been thoroughly studied. Other senomorphics are plant extracts consisting of a mixture of terpenes, alkaloids, and polyphenols. The biological effects of these compounds are multiple, ranging from the activation of antioxidant enzymes to the reduction in interleukin or MMP expression, and the inhibition of MAPKs. Data in Table 2 show that most senomorphics modulate the senescent phenotypes to disrupt the proinflammatory nature of senescent cells.

**Table 2:** Senomorphics that block SASP components at the secreted level only (measured by ELISA, antibody arrays, and multiplex array). Arrows mean that the secretion of all written factors is decreased upon treatment with the corresponding compound, compared to untreated senescent cells.

Compound	Function	Cell Type	Inducer	Effect on SASP Factors	References
Adalimumab (monoclonal antibody)	TNFα inhibitor	HUVECs	Replicative senescence	IL-6 ↓	[178]
Anti-ephrin B2 antibody (clone B11)	Ephrin B2 inhibitor	Human fibroblasts	Chemical-induced senescence Irradiation-induced senescence	IL-6 ↓	[179]
Apigenin (flavonoid)	NF-κB inhibitor	BJ fibroblasts	Bleomycin-induced senescence	IL-6; IL-8; IL-1β ↓	[183]
Avenanthramicine C	AMPK activator p38/NF-κB inhibitor	Human fibroblasts (HDFs)	Replicative Senescence	IL-6; IL-8; TGF-β ↓	[184]
BIRB796	p38 inhibitor	Human fibroblasts (NHDFs)	Replicative Senescence	IL-6 ↓	[185]
Cortisol/corticosterone	Glucocorticoids	Human fibroblasts (HCA2)	X-irradiation induced senescence	IL-6 ↓	[186]
Hydroxytyrosol (olive phenolic compound)	NF-κB inhibitor	Human fibroblasts (NHDFs , MRC5)	Replicative senescence	IL-6; MMP-2; MMP-9 ↓	[187]
IPI-504	HSP90 inhibitor	ARPE-19	H_2_O_2_-induced senescence	IL1-β; IL-8 ↓	[188]
Isatis tinctoria L. Leaf extract (ITE)	mTOR/MAPK/NF-κB inhibitor	Human fibroblasts (HDFs)	Replicative Senescence	IL-6; IL1-β; IL-8 ↓	[189]
Kaempferol (flavonoid)	NF-kB inhibitor	BJ fibroblasts	Bleomycin-induced senescence	IL-6; IL-8; IL-1β ↓	[183]
Lamivudine	Nucleoside reverse transcriptase inhibitor	Human fibroblasts	Replicative Senescence	IFN-1 ↓	[190]
Metformin	Several pathways	Human HNSCC cell line Cal27	LY2835219 (CDK4/6 inhibitor)- induced senescence	NT3; MCP-1; IL-6; IL-8; GRO; IGFBP1; BMP4; BLC ↓	[191]
Metformin	Several pathways	Primary VSMCs from the aortas of elderly patients	Ang II-induced premature senescence	MMP-2; IL-6; TGFβ ↓	[192]
Mix of bioCurcumin, Polydatin and liposomal-b-caryophyllene	Several pathways	HUVECs	Replicative Senescence Doxorubine-induced senescence	IL-6; IL-1β ↓	[193]
MK2.III	MK2 kinase inhibitor	Human fibroblasts (NHDFs)	Replicative Senescence	IL-6 ↓	[185]
Oleuropein (olive phenolic compound)	NF-κB inhibitor	Human fibroblasts (NHDFs) MRC5	Replicative senescence γ-irradiation-induced senescence	IL-6; MMP-2; MMP-9↓ IL-6; IL-8; MCP-1; RANTES ↓	[187,194]
Simvastatin	HMG-CoA reductase inhibitor	Normal Human Fibroblasts (HCA2)	γ-irradiation-induced senescence	IL-6 ↓	[195]
Rapamycin	mTOR inhibitor	Normal Human Fibroblasts (HCA2)	γ-irradiation-induced senescence	IL-6; CSF2; CCL7; CCL8; IGF1; TGFB3; IL-8; BMP4; IL-10 ↓	[196]
Rapamycin	mTOR inhibitor	Murine MEFs	H_2_O_2_-induced senescence	TNFα; LIX; Leptin R; MIP-1α	[197]
Resveratrol	SIRT1 activator NF-κB inhibitor NRF2 activator	Arterial VSMCs derived from aged rhesus monkeys	Chronological age	MCP-1; TNFα, VEGF	[198]
Ruxolitinib	JAK1/2 inhibitor	Preadipocytes from healthy human kidney transplant donors	γ-irradiation-induced senescence Replicative senescence	IL-6; GM-CSF; G-CSF; IL-10; CXCL-1; MIP-1a; IL-8, MCP-1; RANTES, MCP-3; PAI-1; MIP-1β; TNFα; IFN-α2; IL-1α; VEGF; CCL-11; PDGF-AA IL-6; IL-8; MCP-1; PAI-1 ↓	[83]
SB203580	p38 inhibitor	Human fibroblasts (NHDFs) Normal Human Fibroblasts (HCA2) Normal Human Fibroblasts (HCA2)	Replicative Senescence γ-irradiation-induced senescence Ras-induced senescence	IL-6 ↓ GRO; IL-6; IL-8; MCP-2; MCP-1; GCP-2; GM-CSF; IL-10; GDNF; IGFBP4, CNTF; GROα; TGF-β1, Angiogenin; IL-2; Eotaxin; IL-7; MIG; IL-1α; TNFα; IL-5; TNFβ; Sgp130; Osteoprotegerin IL-6; IL-8; GM-CSF ↓ GM-CSF; IL-6; GRO; MIP-1α; IL-1β; ENA78; GROα; IL-8; MCP-3; HGF; ICAM3; MIP-1β; uPAR; Dtk; IGF-1SR; IL-1α; Sgp130; IL-12 p40; IL-4; TIMP1; IL-11; PIGF; IL-15; IL-2; RANTES; IL-2 Rα; Oncostatin M; GDNF; MIP-3α; IL-12 p70; Thrombopoietin	[89,185]
Silybum marianum flower extract (SMFE)	Unknown	Human fibroblasts (HDFs)	Replicative Senescence	IL-6; MMP-1 ↓	[199]
SR9009	Reduces ROS level via the activation of the NRF2 pathway	Human fibroblasts (HDFs)	Doxorubicin-induced senescence	IL-1α; IL-1β ↓	[200]
UR-13756	p38 inhibitor	Human fibroblasts (NHDFs)	Replicative Senescence	IL-6 ↓	[185]
Wogonin (flavonoid)	NF-κB inhibitor	BJ fibroblasts	Bleomycin-induced senenscen	IL-6; IL-8; IL-1β ↓	[183]
Zileuton	5-LO inhibitor	Human fibroblasts (HDFs)	γ-irradiation-induced senescence	IL-6 ↓	[201]

Most studies, however, have only assessed a few SASP major factors (such as IL-6, IL-1β, and MMPs) following senomorphic treatments, which is not representative of SASP as a whole. Moreover, the impact of senomorphics on the secretion of ECM components, microvesicles, and complex lipids remains largely unexplored. Senomorphics can act on multiple targets depending on the context, the nature, and the model of senescence. In some cases, we cannot rule out that they might even increase the secretion of some detrimental factors. This raises the concern that SASP resulting from senomorphic treatment should probably be less deleterious and should be considered as modified rather than non-senescent-like. In addition, few studies are using CM from senescent cells treated with senomorphics to examine the biological effects of the modified SASP (such as the pro-tumoral impact or differentiation) on other cell types. A study demonstrated that CM from senescent HUVECs treated with anti-TNFα reduced the migration and mammosphere formation of MCF7 cells compared to the CM obtained from untreated senescent HUVECs [178]. Other authors showed that CM from senescent preadipocytes treated with a JAK inhibitor (ruxolitinib) prevented macrophage migration, reduced inflammation in non-senescent preadipocytes [83], and attenuated osteoclast differentiation [202]. CM collected from simvastatin-treated senescent fibroblasts suppressed breast cancer cell proliferation by reducing the phosphorylation of ERK1/2 [195]. Furthermore, CM of senescent cancer cells treated with metformin attenuated the sphere-forming ability of growing cancer cells [191]. Metformin has also been shown to reverse the decrease in the migration of senescent VSMCs [192]. Therefore, in the absence of more extensive data, it is difficult to assess the real effectiveness of senomorphics on SASP.

## 7. Conclusions and Future Directions

**Figure 2:**
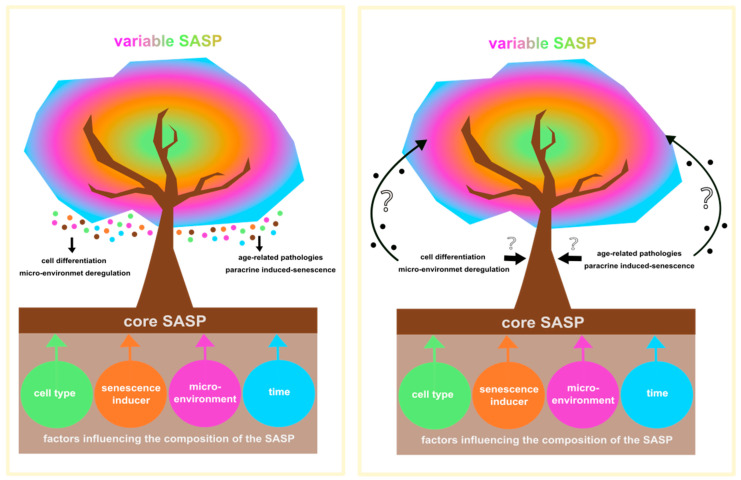
The crosstalk between the senescent cells and their microenvironment through the SASP. The core SASP is defined as the common SASP components shared between different senescent cells regardless of the cell type, senescence inducer, micro-environment, or time-point. The final composition of SASP (colored dots) is the combination of the core SASP and the variable SASP, which is defined by the four different factors mentioned above. Altogether, the whole SASP will influence different physiological and pathophysiological processes, such as cell differentiation, micro-environment deregulation, paracrine induced-senescence, and age-related pathologies (**left** panel). However, the question remains, are those modulations of the cellular environment influencing, in turn, the composition of SASP, and to what extent the core and variable SASP are affected (**right** panel)?

Despite the efforts to characterize the senescence phenotype and the composition of SASPs, understanding the biological effects at a given time for a given cell type and senescence inducer remains challenging. Senescent cells are very heterogeneous, therefore, comparing the effects and status of senescence between senescent cells is a complex operation. Indeed, senescence evolves based on the microenvironment and time elapsed. This modulation suggests that the factors responsible for initiating senescence may not be the same as those involved in maintaining the senescent phenotype. In other words, the “initial/early” senescence is not the same as the “terminal/late” senescence. Once senescence is reached, senescent cells can correct some characteristics of their phenotype via the microenvironmental context. This indicated that the tissue’s physiological and pathological context contributes to the extent, intensity, and regulation of senescence. In turn, the new information received can prompt senescent cells to adapt and produce an SASP that is more attuned to the context, meaning that the communication of the senescent cells is not unidirectional (Figure 2). This gives rise to a large variety of SASPs, and the different combinations of SASP components could generate distinct « barcodes » that may dictate SASP-mediated responses. For this reason, machine learning might be a promising tool to help the better characterization of SASPs [203]. 

Although efforts have been made to understand the effects of secreted factors, particularly, pro-inflammatory factors, little is known about the effects of changes in the ECM as well as direct cell-to-cell contacts. Secreted factors are only one of the means by which cells communicate with their matrix and cellular microenvironment [13]. Moreover, most studies on senescence have been carried out on fibroblasts, a model that, in essence, does not give rise to direct intercellular communication. Furthermore, the precise role of each class of secreted molecules in mediating the biological effects of SASP remains unclear. While the pro-inflammatory portion is known to be related to the pro-oncogenic effects of SASP, the role of secreted matrix proteins is not well understood. Models of decellularized matrices from senescent cells could help to fill this gap in our knowledge.

Beyond the fact that SASP may evolve and that we lack knowledge on temporal aspects, data on the transcriptomic, proteomic, and secretomic concordance are necessary. There are limited secretomic data available to support conclusions about the effects of SASP. In addition, co-culture models could be of great interest for modeling cellular interactions and the evolution of SASP, as the presence of other cell types can influence the secretome of senescent cells, either by slowing it down or stimulating its production. Several studies have attempted to define a core SASP, which is very useful to understand the common aspects following different senescence inducers or cell types. However, from a therapeutic point of view, it would be more interesting to understand what differentiates two cell types that have undergone the same senescence inducer. As an example, when two SASPs have a 70% similarity, it raises questions about the potential impact of the remaining 30% difference. This highlights the potential for the selective use of SASP blockers. Regarding SASP blocking molecules, their senomorphic properties have been essentially based on their ability to inhibit the transcription of SASP genes. However, concordance between transcriptomics, proteomics, and secretomics has not been demonstrated for all SASP genes. Further experiments are needed to validate this concordance as well as data using CM from senescent cells treated with senomorphics. 

In conclusion, there is still much to be discovered regarding how SASP communicates with its environment and network, and it is only through multidisciplinary approaches that we can better understand and decode them. Finally, it is still challenging to critically evaluate the use of SASP in vivo, as modulating its components has demonstrated limited reliability on human health. To be continued in the next “What SASP” discussion.

## Figures and Tables

**Table 1:** Common SASP proteins shared between different cell types under different inducers of senescence.

SASP Factor	Cell Type	Inducer	Detection	References
IL-6	Human fibroblasts (WI-38, IM90, BJ) Prostate epithelial cells (PrECs) Human keratinocytes (NHEK)	Irradiation-induced senescence Replicative Senescence Ras overexpression UVB-induced senescence	Antibodies array ELISA	[9,29,70]
CXCL1-2-3	Human fibroblasts (IMR90, HCA2) Prostate epithelial cells (PrECs)	Irradiation-induced senescence Replicative Senescence	Antibodies array	[9,29]
IL-8	Human fibroblasts (WI-38, IM90, BJ), Prostate epithelial cells (PrECs) Renal epithelial cells (ATCC) Human keratinocytes (NHEK)	Irradiation-induced senescence Replicative Senescence UVB-induced senescence Glyoxal-induced senescence	Antibodies array Mass spectrometry ELISA	[9,29,34,60,70]
IGFBP-2	Human fibroblasts (IMR90, WI-38, HCA-2, BJ) Renal epithelial cells (ATCC)	Irradiation-induced senescence	Antibodies array Mass spectrometry	[29,60]
IL-7	Human fibroblasts (WI-38, HCA-2, BJ) Prostate epithelial cells (PrECs)	Irradiation-induced senescence	Antibodies array	[9]
GDF15	Human fibroblasts (IMR-90) Epithelial renal cells (ATCC)	Irradiation-induced senescence Replicative Senescence	Mass spectrometry	[60]
Macrophage migration inhibitory factor (MIF)	Human fibroblasts (IMR-90, WI-38) Epithelial renal cells (ATCC) Human keratinocytes (NHEK) Prostate epithelial cells (PrECs) Bone marrow MSC	Irradiation-induced senescence UVA-induced senescence RAS overexpression Chemical-induced senescence (ATZ) H_2_O_2_-induced senescence	Mass spectrometry Antibodies array	[9,60,71,72]
Filamin B	Human fibroblasts (IMR-90) Epithelial renal cells (ATCC) Human keratinocytes (NHEK) Prostate epithelial cells (PrECs) Bone marrow MSC	Irradiation-induced senescence UVA-induced senescence RAS overexpression Chemical-induced senescence (ATZ) H_2_O_2_-induced senescence	Mass spectrometry	[9,60,71,72]
Cathepsin D	Mesenchymal stem cells	Replicative Senescence Chemical-induced senescence (doxorubicin) H_2_O_2_-induced senescence Irradiation-induced senescence	Mass spectrometry	[72]

## Data Availability

The data presented in this study are available in the article.
